# 

**Published:** 1868-08-01

**Authors:** 


VOL. XXV. NUMBER 15.

### THE CHICAGO MEDICAL JOURNAL.

*PUBLISHED SEMI-MONTHLY.*

J. ADAMS ALLEN, M.D., LL.D.
EDITOR.
WALTER HAY, A. M., M. D.
ASSOCIATE EDITOR.

AUGUST 1, 1868.

CHICAGO:
CHURCH, GOODMAN AND DONNELLEY, STEAM BOOK AND JOB PRINTERS,
108 AND 110 DEARBORN STREET.

